# Scrutinizing mechanical circulatory support in cardiogenic shock: Have we jumped the gun?

**DOI:** 10.1186/s13054-024-04853-y

**Published:** 2024-03-15

**Authors:** Enzo Lüsebrink, Hugo Lanz, Holger Thiele

**Affiliations:** 1 Department of Medicine I, LMU University Hospital, LMU Munich, Marchioninistraße 15, 81377 Munich, Germany; 2 DZHK (German Center for Cardiovascular Research), Partner Site Munich Heart Alliance, Munich, Germany; 3 Department of Internal Medicine/Cardiology and Leipzig Heart Science, Heart Center Leipzig at University of Leipzig, Strümpellstr. 39, 04289 Leipzig, Germany

**Keywords:** V-A ECMO, Impella, Mechanical circulatory support, Cardiogenic shock, Myocardial infarction

## Abstract

Despite increasing therapeutic options and disposable resources, cardiogenic shock (CS) remains a formidable condition with high mortality. Today, veno-arterial extracorporeal membrane oxygenation and microaxial flow devices (Impella, Abiomed, Danvers, USA) are established forms of mechanical circulatory support (MCS) in CS, with increasing application over the years. Despite this trend, incorporation into current ESC (Class IIa, evidence C) and AHA/ACC (Class IIa, evidence B-NR) guidelines is based nearly exclusively on observational results. Despite these recommendations and increasing application, current evidence from randomized controlled trials has not provided clear mortality benefit. Thus, reflection on current evidence is hereby justified.

## Evidence V-A ECMO

Results from small RCTs have failed to show mortality benefit of V-A ECMO in acute myocardial infarction (AMI)-CS [1]. The ECLS-SHOCK trial recently added to the evidence by providing the largest V-A ECMO RCT to date, failing to show mortality improvement in 420 (AMI)-CS patients [2]. Despite a large sample size, limitations to this RCT and preceding studies exist: (1) Open-labeled design of the ECLS-SHOCK trial may have affected decisions of treating physicians when implementing MCS, (2) high cross-over rates and subsequent delay of V-A ECMO initiation in control patients may weaken potential benefit, (3) cross-over to alternative MCS devices in control arms does not justify calling results a comparison against “medical-therapy alone”, as this likely reflects common real-world practice, in which treatment strategies such as inotropic support or different MCS devices must be adapted according to patient characteristics, and (4) despite need for pragmatic inclusion criteria in RCTs, comparing patients receiving mandatory MCS use once inclusion criteria are met with patients receiving medical therapy that receive MCS support, when deemed useful, is a limitation across all trials.

Further, a recent individual patient data meta-analysis incorporating 567 AMI-CS patients from four RCTs showed no significant 30-day mortality benefit for V-A ECMO (45.7%) versus medical-therapy (47.7%) (OR 0.93; 95%CI 0.66–1.29) [3]. Given the above-mentioned mortality outcomes, evidence regarding the following aspects must be scrutinized: (1) Do specific subgroups benefit from V-A ECMO? Current guidelines do not reflect upon patient selection to guide MCS initiation. Subgroup analyses from the above-mentioned meta-analysis, assessing age (> 65 vs. ≤ 65y), sex (male vs. female), lactate (< 5 vs. ≥ 5 mmol/l), cardiac arrest, type or location of infarction (STEMI vs. NSTEMI, anterior vs. other) as well as post-PCI results (TIMI 0/1 vs. 2/3), provided no survival benefit [3]. (2) Does timing of V-A ECMO influence outcomes? Observational results suggesting benefit of initiation pre-PCI were refuted in the ECLS-SHOCK trial, where roughly 50% implementation prior or during revascularization provided no mortality benefit [2]. (3) Do current safety outcomes following V-A ECMO justify application? Across all studies, moderate or severe bleeding occurred more often in V-A ECMO versus control patients (OR 2.44; 95%CI 1.55–3.84) [3]. This indicates that guideline directed therapy may be harmful, as bleeding in AMI-CS leads to worse outcomes. Further, higher peripheral ischemic complication rates were seen in V-A ECMO versus control patients, despite high antegrade perfusion cannulae application (OR 3.53; 95%CI 1.70–7.34) [3]. Lastly, scrutiny of V-A ECMO modification such as left ventricular (LV)-unloading via V-A ECMO + Impella (ECMELLA) or V-A ECMO + intra-aortic balloon pump (IABP) is warranted due to associated increase in bleeding or hemolysis. Thus, V-A ECMO is associated with serious complications that worsen outcomes, making future selection based on solid evidence essential. While awaiting randomized evidence for mortality benefit of V-A ECMO, increasing application and high complication rates justify questioning the validity of this approach.

## Evidence Impella

Since IABP application decline in CS following guideline recommendation downgrade, use of Impella—like V-A ECMO—has steadily increased. Despite this trend, sound evidence assessing benefit is limited to few small RCTs. In a meta-analysis including four RCTs comparing MCS (two TandemHeart and two Impella) with IABP (control), the ISAR-SHOCK and IMPRESS in Severe Shock trials found no short-term mortality difference following Impella vs. IABP (RR 1.01; 95%CI 0.70–1.44, *p* = 0.98, *I*^2^ = 0%). Also, increased bleeding and a numerically higher incidence of limb ischemia following MCS were reported [4–6]. Larger propensity-matched studies have shown similar or even higher in-hospital mortality and increased major bleeding following Impella compared to IABP. One recent large observational adjusted study comparing 23,478 AMI-CS patients receiving Impella versus alternative treatments again found higher Impella-associated 30-day mortality across multiple analyses [7].

As with V-A ECMO, these results beg the question of clinical benefit in certain subgroups. Unfortunately, lack of meaningful subgroup analyses in the above-mentioned studies clouds the question of which patient (if any) will prove to be the “optimal” Impella candidate. As data from larger RCTs such as DanGer Shock (NCT01633502) are eagerly anticipated, evidence providing clear advantages of Impella remains non-existent. Until available, indications of growing Impella use despite higher complication rates without mortality benefit should be alarming, forcing us to question which (if any) future role it will play in CS management.

## Future application of MCS in CS: is there still a spot at the table?

Management of CS beyond initial V-A ECMO or Impella implementation binds many resources and requires experienced multidisciplinary teams. Both efficacy and safety outcomes demonstrate that a “one-size fits all” application in AMI-CS finds no justification, and thus the question of whether we are harming patients with current guideline recommendations remains. Lessons can be taken from the past, as it was not until RCTs proved a lack of mortality benefit of commonly used IABP in AMI-CS that forced a recommendation downgrade in ESC guidelines (Class III, level B). Thus, the question remains: Does MCS provide a mortality benefit, and if yes, in which CS subgroup? Surely, if existent, this subgroup will likely be small (< 10% of all CS patients) considering high mortality rates of 40–50% across all CS patients [3].

With stagnating CS mortality rates, high resource requirements, and increasing number of patients requiring hemodynamic stabilization, finding the right therapy for the right patient will become increasingly difficult. Though questioning MCS in other clinical scenarios would be premature, results from the upcoming randomized DanGer Shock trial, which excluded patients with out of hospital cardiac arrest (OHCA), may shed light on whether patients without OHCA benefit from MCS. Until then, application in patients with OHCA should be restricted due to low likelihood of mortality benefit. Until further RCTs provide better understanding of MCS-associated benefits, complications, and management, this costly therapy should be allocated to a very small group of patients with foreseeable survival and reasonable long-term prognosis.

## Data Availability

Not applicable.
